# Diseases of the Nervous System

**Published:** 1881-08

**Authors:** 


Diseases of the Nervous System.—Being a treatise on Spasmodic, Paralytic, Neuralgic and Mental Affections. For the use of Students and Practitioners of Medicine. By *Charles Porter Hart,*

*M. D.*, author of "Repertory to New Remedies," etc., etc. pp. 409, price $3.00. Boericke & Tafel: New York and Philadelphia. London: Trübner & Co., Ludgate Hill. Homœopathic Publishing ing Co., 2 Finsbury Circus. 1881.

The diseases discussed in this work of Dr. Hart's, are among the most difficult chronic cases which the physician is called upon to treat. Being most difficult, the physician needs the best advice to cope successfully with them. This he will ever find by a rigid adherence to our *law;* in mild, simple cases, one might, without danger, deviate from it, but in difficult chronic cases, any deviation means failure.

We are apt to be frightened by the terrible *names* of paralysis, epilepsy, hysteria, etc., and to think our minute doses and single remedies too simple, and insufficient to cure them. These diseases are very often incurable under any treatment; but why desert our law which does offer *some* hope, for allopathic measures that have failed in the hands of their experts? To do so, is irrational.

Our author commences his task by a short *résumé* of the physiology of the cerebro-spinal centres, rightly deeming a clear understanding of the physiology of these parts necessary to any rational study of their diseases.

Then follow chapters on the derangement of the motor functions—convulsions, epilepsy, chorea, tetanus, etc.; next are described the paralytic disorders —paralysis in general, hemiplegia, paraplegia, infantile, facial, diphtheritic and other paralyses, muscular atrophy, etc.; derangement of sensory function follows next, and under this head we find neuralgias, general and special, angina pectoris, gastralgia, spinal irritation, etc.; lastly, we have moral and mental disorders.

The descriptions given of these numerous diseases are short, but in the main clear and sensible; the field embraced is one not well explored at present by physiologist or pathologist, hence, many points are yet unknown or undecided. As to treatment, our author has given the remedies most frequently prescribed under each disease, and in many instances, the symptoms of the remedy are illustrated by clinical cases. These cases are culled from all sources, and hence, some are of dubious character and under our law, useless, but in the main they are good.

The work is one that will repay perusal, and will be of assistance in many tedious chronic cases.

